# Epidemiology of injuries among U18, U19, U21 and senior elite netball players

**DOI:** 10.17159/2078-516X/2020/v32i1a7577

**Published:** 2020-01-01

**Authors:** CJ Sinclair, FF Coetzee, R Schall

**Affiliations:** 1 Department of Exercise and Sports Sciences, Faculty of Health Sciences, University of the Free State, Bloemfontein, South Africa; 2 Department of Mathematical Statistics and Actuarial Science, Faculty of Natural and Agricultural Sciences, University of the Free State, Bloemfontein, South Africa

**Keywords:** incidence, sport high performance, injury profile

## Abstract

**Background:**

Despite a substantial body of literature on injuries among elite netball players in South Africa, no study reports on the timing and type of injuries and the reason for injuries.

**Objective:**

To determine the epidemiology of injuries in U18, U19, U21 and senior netball players in the Free State (FS), South Africa, over two consecutive netball seasons (2017/2018).

**Methods:**

An injury questionnaire was used to collect data on 96 eligible players.

**Results:**

A total of 48 injuries were reported. The profile of injuries revealed that 58% (n=28) of the injuries occurred during matches, 29% (n=14) during practice and 13% (n=6) during preseason training. Acute injuries accounted for 54% (n=26) of the total, while 46% (n=22) were overuse injuries. A third of all the injuries were re-injuries. The centre (C) position had the highest incidence of injuries in players (n=14; 29%). The ankle was the most frequently injured body part (n=18; 36%), followed by the lower leg and Achilles tendon (n=6; 13%) thus largely the ligaments and muscles. The overall incidence rate of injuries during match play was 33.9 injuries per 1 000 hours of match play.

**Conclusion:**

Preventative strategies should consist of ankle and lower leg strengthening and neuromuscular balance techniques. The focus should be on correct landing techniques, results of abrupt change of direction movements and short bursts of speed.

Netball is a dynamic and physically demanding sport involving high-intensity movements, such as jumping, breaking/stopping, lunging and leaping, interspersed by periods of low-intensity movements, such as walking, standing and passing the ball. The combination of high- and low-intensity movements can predispose players to lower limb injuries, as the demands on athletes are physically and mentally challenging.^[1,2]^

Studies have indicated that the most frequently injured joints in netball are the ankle and knee, of ligamentous or musculoskeletal anatomical origin.^[1–5]^ Furthermore, Hopper reported that the prevalence of lower limb and back injuries might account for 23% of injuries in netball players.^[4]^

Previous researchers have studied possible risk factors for injury, such as hypermobility of joints and somatotyping.^[2–4,6,7]^ Other factors, such as poor landing techniques, fast rotational movements and contact with other players, could also be regarded as predisposing factors for injuries.^[5,6]^ A study by Pillay and Frantz^[8]^ concluded that the lack of a warm-up before a game, training for four or more hours a week, and being injury-free within the past 12 months decreased the risk of injury. Coetzee et al.^[9]^ found that the playing surface has an impact on injury, for example, cement surfaces accounted for 80% of knee injuries as opposed to 20% on synthetic surfaces. The majority (89%) of serious injuries occurred on cement surfaces.^[9]^

However, limited data on netball injuries in South Africa is available, and only at senior level or during match play at a specific tournament.^[1,8]^ Data on the epidemiology of injuries can contribute to the development of programmes for the prevention of injuries. In this study, the injuries recorded were those received during the preseason, practice and match play during two consecutive seasons in four different age categories (U18A high school league, U19, U21 and senior) in the Free State (FS), South Africa. Detailed information on the incidence of injuries by age group, player position, anatomical site and time of play was collected. To these authors’ knowledge, this is the first such study in South Africa on the epidemiology of injuries in netball players in four age groups.

Therefore, the objective of this study was to determine the epidemiology of injuries in U18, U19, U21 and senior FS netball players. Specifically, the study was conducted to report data on the following aspects of injuries in netball players:

- the incidence of injuries by age group;
- the number and proportion of injuries by player position, namely goal attack (GA), wing attack (WA), goal defence (GD), goal shooter (GS), centre (C), wing defence (WD) and goalkeeper (GK), as well as the number and proportion of injuries by anatomical site in the various age groups;
- the type of injuries in the various age groups;
- the number and proportion of injuries during play times (match versus practice injury, and the playing quarter for match injuries) per age group.

## Methods

A cross-sectional, descriptive study was conducted to determine the epidemiology of injuries among U18A league secondary school players, U19, U21 and senior netball players in the Free State during the 2017 and 2018 seasons. The total study population consisted of 96 players (U19, U21 and senior n=12 each, and n=60 U18A league secondary school players).

An injury was defined as any physical complaint sustained by a player during or following a netball game or practice that required medical treatment, associated with either loss of time or performance restriction.^[10]^ An injury questionnaire was used to collect data on the nature of all injuries during the study period. The questionnaire was based on the consensus statement on injury definitions in rugby union^[10]^ and adapted for netball.^[1]^

When an injury was sustained, it was diagnosed by a medical doctor. All players from all teams were approached weekly as a group to determine who had sustained an injury the previous week and attained a medical diagnosis. After the player signed the informed consent form, a research assistant helped the player to complete the questionnaire pertaining to the injury. When players played more than one position, the player’s position was recorded based on the position they were playing at the time of the injury

Data analyses were done using SAS Version 9.4 (SAS Institute Inc., Cary, NC). The number and percentage of injuries were determined with regard to their timing (during a match, preseason, practice), type (acute/trauma, overuse, re-injury), how the injury was sustained (possible contact with a player), per player position, per anatomical site of injury, and severity of injury. Injuries were categorised as follows: slight (0 days absent), minor (1–7 days absent), moderate (8–28 days absent) or major (more than 28 days absent).

The injury incidence rate was calculated as the number of injuries per 1 000 playing hours, where a playing hour refers to an individual playing for one hour (thus a match lasting one hour represents 14 playing hours for the two participating teams).

The study was approved by the Health Sciences Research Ethics Committee (HSREC) of the University of the Free State (UFS-HSD 2017/0014). Permission was obtained from the South African Netball Association and FS Netball, and the FS Department of Education, the headmasters of secondary schools participating in the U18A netball league, netball coaches and finally, parents if the player was younger than 18 years of age, and the players themselves.

## Results

### Profile of injuries

A total of 48 injuries were reported of which eight were senior players, five U21, six U19 and twenty-nine U18A players. Table 1 indicates the overall profile of injuries as the number of injuries per age group. In addition, in this study, 16 (33%) injuries were re-injuries and 22 (46%) overuse injuries. Furthermore, the timing and nature of the injuries and how the injuries occurred are summarised.

### Injuries by player position

Table 2 presents the injuries by player position and age group, and overall by player position. Overall, the C 14 (29%) sustained most injuries, followed by the GA 12 (25%) and WD 9 (19%), with the GK 1 (2%) having the lowest incidence of injuries.

### Anatomical site of injuries

Table 3 presents the number and percentage of injuries per anatomical site by age group and overall. Overall, the most frequently occurring injuries involved the ankle joint (n=18; 38%), followed by injuries to the lower leg and Achilles area, face and/or head (n=6; 13%), groin injuries, (n=5; 10%), and injuries to the knee (n=4; 8%).

### Type of injuries

Table 4 presents the type of injuries by age group. The most common injuries were ligament tears/strains (n=17; 36%). Concussion and fractures were recorded only in the U18A group (n=3 each; 6%).

### Time of injury

Table 5 summarises the timing of injuries; for match injuries the number per quarter is presented. Furthermore, the number of preseason injuries and the number of injuries that occurred during a practice session are reported.

Of the 48 injuries, 32 (67%) were match injuries, of which four was sustained in a practice match. Approximately two-thirds of match injuries overall were sustained during the second and third quarters of the match (21 out of 32 match injuries, 67%). Twenty out of the 48 injuries (42%) occurred during practice sessions, preseason or in a practice match.

### Severity of injury

Table 6 displays the severity of injuries by age group and overall. Overall, five (11%) were slight, 12 (26%) minor, 19 (40%) moderate, and 11 (23%) were major injuries.

### Injury incidence per 1000 player hours

Table 7 summarises the number of matches played by each age group, the percentage of injuries that occurred during match play, the total number of playing hours and the incidence of injuries during match play per 1 000 hours. The highest incidence of injuries was observed in the U19 age group (79.4 injuries per 1 000 hours).

## Discussion

### Profile of injuries

The objective of this study was to determine the epidemiology of injuries in U18, U19, U21 and senior FS netball players by reporting detailed data on the incidence of injuries by age group, player position, anatomical site and time of play. The study included four age groups of players and recorded injuries over two consecutive seasons, including injuries sustained during practice and preseason training.

These findings can be compared with relevant research in South Africa,^[1,2,8]^ on the epidemiology of injuries; however, existing studies focused on senior participants, either at a specific tournament, one specific age group, or over a single competitive netball season. A number of international studies evaluated injury incidence, mechanism of injury, incidence by player position and severity of injury for an entire netball season or at a specific netball tournament.^[5,11–14]^

In this study of netball players in the FS U18A high school league, U19, U21 and senior players, an overall injury incidence rate of 33.9 injuries per 1 000 playing hours for match injuries was reported (Table 7). This is similar to the incidence rate reported by Langeveld et al.^[1]^ which was 501 injuries per 1 000 hours of match play, or 35.8 injuries per 1000 playing hours. Botha reported an injury rate of 22.5 injuries per 1000 playing hours in U15 and U16 school children in South Africa.^[15]^

Injuries can be attributed to many factors, such as incorrect landing, slipping, tripping and direct trauma.^[11]^ In this study, 26 injuries (54%) occurred due to trauma of some kind. Furthermore, 24 of the 48 injuries with relevant information (50%) resulted from contact with another player. This proportion of contact injuries is notably high when considering the rules of netball, as contact is prohibited, and a penalty is (or should be) awarded to and/or a warning issued to the opponent in such cases.

However, in the present study, the detailed causes or presumed causes of injuries, such as landing incorrectly, slipping and tripping, were not recorded. McManus et al.^[11]^ found that 38% of injuries to the knee and ankle could be attributed to incorrect landing techniques. Furthermore, McManus et al. ^[11]^ found that slipping, tripping and landing incorrectly while moving forward were the main causes of injuries, although they did not consider contact with another player as a reason for injury. Similarly, Pillay and Frantz^[8]^ in their study on South African netball players found that 19% of injuries at the knee region and 29% to the ankle could be attributed to incorrect landing techniques. Ellapen et al.^[2]^ investigated non-elite netball players in KwaZulu-Natal (KZN) and obtained similar results, with incorrect landing techniques contributing to 63% of ankle injuries and 57% of knee injuries. They did, however, consider contact with another player as a reason for injury, but this accounted for only 21% of all of the injuries in their study.^[2]^

Additionally, in the present study, injuries were recorded by body segment and which body region (left, right or bilateral) was injured. The right side had 25 (52%) injuries compared to 12 (26%) on the left side. This is similar to findings by Langeveld et al.^[1]^ who found that ankle/foot injuries occurred on the right side 59% of the time compared to 41% on the left side. Knee injuries occurred on the right side 53% of the time and 47% on the left side.^[1]^ In this present study a total of 11 (23%) bilateral injuries were observed, which could be attributed to overuse injuries to the lower leg, such as medial tibial stress syndrome, or to the lower back such as muscle spasms and triggers. In certain sports, the dominant leg may be at increased risk of injury because it is preferentially used for kicking, pushing off, jumping, or landing. However, the association between limb dominance and injury is controversial.^[18]^ One found that left-leg dominant athletes were more likely to sustain an ankle injury. Another study reported that the dominant leg sustained more non-contact knee injuries and one found an increased incidence of ankle injuries on the dominant side, while three other studies reported no association between limb dominance and injury. Therefore, the relationship between limb dominance and injury remains unclear.^[18]^

### Injuries by player position

This present study’s data (Table 2) show that the C, GA and WD have the highest risk of injuries, while the GK has the lowest. This is similar to the findings of Smith et al.,^[7]^ who reported that GA, WA and GD suffered the most injuries and GK the least. Similarly, Hopper et al.^[6]^ found that the WA, C and GS had an increased incidence of injury, while the GK showed minimal risk. In contrast, McManus et al.^[11]^ reported that defensive injuries (22.8% by either the GK or GD) were more likely to occur than any other injuries elsewhere on the court. The higher risk of injury for the C, GA and WD found in this present study can possibly be explained by the higher physical demands on these players during match play.

### Anatomical site and type of injuries

Regarding the anatomical site of injuries (Tables 3 and 4), the overall occurrence was 18 (36%) ankle injuries, followed by six injuries (13%) to the lower leg and Achilles area. Previous research also reported a high incidence of ankle and, contrary to the present study, knee injuries.^[1,2,5–7]^ Of the 22 (46%) overuse injuries (Table 1), six injuries were to the lower leg and Achilles area, which were mainly bilateral and affected overloaded areas.

The most prevalent types of injuries were ligament and muscle strains. This finding was in agreement with the results reported from a number of other studies.^[1,2,5–7]^

### Time of play when the injury occurred

Of the 48 injuries reported in the study (Table 5), 30 (63%) occurred during matches, while 20 (42%) took place during either the preseason (n=6; 13%) or practice (n=14; 29%). Of the 28 match injuries, the majority occurred either in the second (n=10; 36%) or the third quarter (n=10; 36%) of matches.

Information on the time of injury might indicate whether an injury could be attributed to fatigue during the match, lack of conditioning in the preseason, or incorrect warmup techniques during the practice session. Only one South African study reported in the literature indicated time of play when the injury occurred with similar results to this study’s findings.^[1]^ No literature could be found on studies reporting injuries in the preseason or during practice, as in the present study.

### Severity of injuries

Most injuries (Table 6) were either minor (n=12; 26%) or moderate (n=19; 40%). This is similar to findings by McManus et al. ^[11]^ who found that 43% of injuries were Grade 2 moderate injuries and 44% were Grade 1 minor injuries. On the contrary, according to the classification of injuries used by Langeveld et al. ^[1]^ the majority of injuries to the ankle joint (42%) were classified as severe (7 days absent). However, the majority (71%) of all injuries in that study were otherwise minor, as the player could return to play by the next game.^[1]^ In the present study, injuries where the player could return to play by the next game were classified as minor injuries, due to the short absence from the game.

### Practical application

This study provided data on the epidemiology of injuries in senior, U21, U19 and U18A elite netball players in the Free State by age group, player position, anatomical site, type, time of play and severity of the injury.

Based on these results and data from the literature, attention should be directed at training modalities that include neuromuscular control and complex training to improve the correct landing techniques during explosive movements,^[14,16]^ in order to prevent injuries sustained during netball. Additionally, short bursts of speed with quick stop-and-go movements, combined with agility and quick change of direction while moving forward, is relative to maximal strength training.^[17]^

Strength and conditioning coaches can use these findings to develop specific conditioning programmes.

## Conclusion

Precautionary measures should target, in particular, the prevention of ankle and knee joint injuries. The inclusion of prehabilitation exercise programmes as part of conditioning at the start of the season may decrease the rate of acute injuries. Additionally, the education of coaches with regard to periodisation of the netball training season and the design of netball conditioning programmes are needed to reduce the relatively high number of overuse injuries found in this study.

The limitations of this study include the small sample size, the use of a questionnaire, which means there could have been underreporting of injuries or inaccurate reporting of the type of injury. Discrepancies were found in the rate of injuries reported in the literature due to different methods of data collection, different definitions of an injury and the interpretation thereof. Furthermore, different netball populations and level of play make comparisons difficult.

## Figures and Tables

**Table 1:** Overall number of injuries by age group for the 2017/2018 netball seasons

Injury profile	Age group				Total

	Senior	U21	U19	U18A	

	n (%)	n (%)	n (%)	n (%)	n (%)
**Total number of injuries**	8	5	6	29	48
**When injury occurred**					
Match play	5 (63)	4 (80)	5 (83)	14 (48)	28 (58)
Preseason	1 (13)	1 (20)	0 (0)	4 (14)	6 (13)
Practice session	2 (25)	0 (0)	1 (17)	11 (38)	14 (29)
**Nature of injury**					
Acute	5 (63)	3 (60)	5 (83)	13 (45)	26 (54)
Overuse	3 (38)	2 (40)	1 (17)	16 (55)	22 (46)
Re-injury (acute or overuse)	3 (38)	2 (40)	2 (33)	9 (31)	16 (33)
**How injury occurred**					
Contact	6 (75)	5 (100)	4 (67)	9 (31)	24 (50)
Non-contact	2 (25)	0 (0)	2 (33)	20 (69)	24 (50)

**Table 2:** Injuries by player position

Player position*	Age group				Total

	Senior	U21	U19	U18A	

	n (%)	n (%)	n (%)	n (%)	n (%)
**Total number of injuries**	8	5	6	29	48
Goal attack (GA)	2 (25)	1 (20)	3 (50)	6 (21)	12 (25)
Wing attack (WA)	2 (25)	0 (0)	0 (0)	2 (7)	4 (8)
Goal defence (GD)	1 (13)	0 (0)	1 (17)	0 (0)	2 (4)
Goal shooter (GS)	1 (13)	0 (0)	1 (17)	4 (14)	6 (13)
Centre (C)	2 (25)	0 (0)	0 (0)	12 (41)	14 (29)
Wing defence (WD)	0 (0)	4 (80)	1 (17)	4 (14)	9 (19)
Goalkeeper (GK)	0 (0)	0 (0)	0 (0)	1 (3)	1 (2)

* Some players play more than one position, but player positions were recorded according to the position they were playing at the time of the injury.

**Table 3:** Anatomical site of injuries

Site	Age group				Total

	Senior	U21	U19	U18A	

	n (%)	n (%)	n (%)	n (%)	n (%)
**Total number of injuries**	8	5	6	29	48
Ankle	4 (50)	4 (80)	2 (33)	8 (36)	18 (38)
Lower leg/Achilles region	0 (0)	0 (0)	0 (0)	6 (21)	6 (13)
Face and/or head	0 (0)	0 (0)	0 (0)	6 (21)	6 (13)
Groin	1 (13)	0 (0)	1 (17)	3 (10)	5 (10)
Knee	1 (13)	0 (0)	0 (0)	3 (10)	4 (8)
Lower back	0 (0)	0 (0)	2 (33)	1 (3)	3 (6)
Feet and/or toes	0 (0)	1 (20)	0 (0)	1 (3)	2 (4)
Forearm and/or wrist	0 (0)	0 (0)	0 (0)	2 (7)	2 (4)
Shoulder and/or clavicle	1 (13)	0 (0)	1 (17)	0 (0)	2 (4)
Hip	1 (13)	0 (0)	0 (0)	0 (0)	1 (2)
Posterior thigh	0 (0)	0 (0)	0 (0)	1 (3)	1 (2)

**Table 4:** Type of injuries sustained

Type of injury	Age group				Total

	Senior	U21	U19	U18A	

	n (%)	n (%)	n (%)	n (%)	n (%)
**Total number of injuries**	7*	5	6	29	48
Concussion	0 (0)	0 (0)	0 (0)	3 (10)	3 (6)
Fracture	0 (0)	0 (0)	0 (0)	3 (10)	3 (6)
Haematoma/bruising	0 (0)	0 (0)	0 (0)	1 (4)	1 (2)
Ligament strain/tear	3 (43)	3 (60.0)	2 (33)	9 (31)	17 (36)
Meniscus/cartilage	1 (43)	0 (0)	0 (0)	0 (0)	1 (2)
Muscle strain/tear/cramp	0 (0)	1 (20.0)	1 (17)	5 (17)	7 (15)
Muscular tendon injury	1 (43)	0 (0)	0 (0)		1 (2)
Other bone injury	0 (0)	0 (0)	0 (0)	4 (14)	4 (9)
Other injury	0 (0)	1 (20.0)	2 (33)	4 (14)	7 (15)
Tendon tear/tendinopathy	1 (14.3)	0 (0)	0 (0)	0 (0)	1 (2)
Tendon tear/tendinopathy & other injury	1 (14.3)	0 (0)	1 (17)	0 (0)	2 (4)

* The type of injury was not recorded for one player in the senior group.

**Table 5:** Time of injury (match or practice)

Time	Age group								Total	

	Senior		U21		U19		U18A			

	Match	Practice	Match	Practice	Match	Practice	Match	Practice	Match	Practice
	n (%)	n (%)	n (%)	n (%)	n (%)	n (%)	n (%)	n (%)	n (%)	n (%)
Preseason period	0 (0)	1 (13)	0 (0)	1 (20)	0 (0)	0 (0)	0 (0)	4 (14)	0 (0)	6 (13)
Practice session	0 (0)	2 (25)	0 (0)	0 (0)	0 (0)	1 (17)	0 (0)	7 (25)	0 (0)	10 (21)
Match play										
1^st^ quarter	1 (13)	0 (0)	0 (0)	0 (0)	0 (0)	0 (0)	0 (0)	3 (10)	1 (2)	3 (6)
2^nd^ quarter	0 (0)	0 (0)	0 (0)	0 (0)	0 (0)	0 (0)	10 (35)	0 (0)	10 (21)	0 (0)
3^rd^ quarter	2 (25)	0 (0)	4 (80)	0 (0)	1 (17)	0 (0)	4 (14)	1 (4)	10 (21)	1 (2)
4^th^ quarter	2 (25)	0 (0)	0 (0)	0 (0)	4 (67)	0 (0)	0 (0)	0 (0)	6 (13)	0 (0)
**Total number of injuries**	5 (63)	3 (38)	4 (80)	1 (20)	5 (83)	1 (17)	14 (49)	15 (53)	28 (58)	20 (42)

**Table 6:** Severity of injuries

Severity	Age group				Total

	Senior	U21	U19	U18A	

	n (%)	n (%)	n (%)	n (%)	n (%)
**Total number of injuries**	7*	5	6	29	48
Slight (0 days absent)	0 (0)	0 (0)	1 (17)	4 (14)	5 (11)
Minor (1–7 days absent)	2 (29)	2 (40)	2 (33)	6 (21)	12 (26)
Moderate (8–28 days absent)	4 (57)	3 (60)	2 (33)	10 (35)	19 (40)
Major (≥ 29 days absent)	1 (14)	0 (0)	1 (17)	9 (31)	11 (24)

* Severity of the injury was not recorded for one player in the senior group

**Table 7:** Number of matches played and rate of incidence of injuries per 1 000 hours

Variable	Age group				Total

	Senior	U21	U19	U18A	
Total number of injuries	8	5	6	29	48
Number of matches played	34	18	9	57	118
Injuries sustained during match play	5 (63*)	3 (60*)	5 (83*)	17 (59*)	30 (63*)
Total playing hours (h)	238	126	63	399	826
Incidence rate (during match play; per 1 000 h)	21.0	31.7	79.4	35.1	33.9

* Percentage of total number of injuries

## References

[1] Langeveld E Coetzee FF Holtzhausen LJ Epidemiology of injuries in elite South African netball players S Afr J Res Sport Phys Educ Recreation 2012 34 2 83 93

[2] Ellapen TJ Schoeman KT Zaca LT Prevalence of musculoskeletal injuries among adolescent recreational netballers in Kwa-Zulu Natal, South Africa British Journal of Medicine and Medical Research 2015 9 3 1 5 Article no. BJMMR. 16966. 10.9734/BJMMR/2015/16966

[3] Hopper D A survey of netball injuries and conditions related to these injuries Aust J Physiother 1986 32 4 231 239 10.1016/S0004-9514(14)60656-5 25025221

[4] Hopper DM Somatotype in high performance female netball players may influence player position and the incidence of lower limb and back injuries Br J Sports Med 1997 31 3 197 199 10.1136/bjsm.31.3.197 9298552 PMC1332516

[5] McMillan JT McLaughlin P Nicholls B An epidemiology of injuries in sub-elite netball during a competitive season: a club perspective [Coursework master’s thesis] Melbourne Victoria University 2004 http://vuir.vu.edu.au/848/ accessed 18 October 2019

[6] Hopper D Elliott B Lalor J A descriptive epidemiology of netball injuries during competition: a five-year study Br J Sports Med 1995 29 4 223 228 10.1136/bjsm.29.4.223 8808533 PMC1332230

[7] Smith R Damodaran AK Swaminathan S Hypermobility and sports injuries in junior netball players Br J Sports Med 2005 39 9 628 631 10.1136/bjsm.2004.015271 16118300 PMC1725309

[8] Pillay T Frantz JM Injury prevalence of netball players in South Africa: the need for injury prevention S Afr J Physiother 2012 68 3 7 10 10.4102/sajp.v68i3.17

[9] Coetzee D Langeveld E Holtzhausen L Training habits, training surface and injuries among South African netball players S Afr J Res Sport Phys Educ Recreation 2014 36 3 39 49

[10] Fuller CW Molloy MG Bagate C Consensus statement on injury definitions and data collection procedures for studies of injuries in rugby union Br J Sports Med 2007 41 5 328 331 10.1136/bjsm.2006.033282 17452684 PMC2659070

[11] McManus A Stevenson MR Finch CF Incidence and risk factors for injury in non-elite netball J Sci Med Sport 2006 9 1–2 119 124 10.1016/j.jsams.2006.03.005 16621712

[12] Partner R Upsall S Francis P Injury epidemiology in female netball players during the 2016/2017 season in the United Kingdom Grad J Sport Exerc Phys Educ Res 2018 9 1 7 http://eprints.leedsbeckett.ac.uk/5136/ accessed 18 October 2019

[13] Hume PA Steele JR A preliminary investigation of injury prevention strategies in netball: are players heeding the advice? J Sci Med Sport 2000 3 4 406 413 10.1016/S1440-2440(00)80007-9 11235006

[14] Hopper AJ Haff EE Joyce C Neuromuscular training improves lower extremity biomechanics associated with knee injury during landing in 11–13 year old female netball athletes: randomised control study Front Physiol 2017 8 883 10.3389/fphys.2017.00883 29163219 PMC5682017

[15] Botha C The epidemiology of injuries in junior netball players in South Africa Master’s dissertation Bloemfontein University of the Free State 2018 https://scholar.ufs.ac.za/bitstream/handle/11660/10045/BothaC.pdf?sequence=1&isAllowed=y accessed 4 April 2020

[16] Humphrys BR Aspe R Clarke R Hughes JD The effects of complex training on neuromuscular development of the lower limbs in youth netball players J Athl Enhanc 2018 7 5 https://www.scitechnol.com/peer-review/the-effects-of-complex-training-on-neuromuscular-development-of-the-lower-limbs-in-youth-netball-players-y8re.php?article_id=8252 accessed 18 October 2019

[17] Thomas C Comfort P Jones PA Dos’Santos T Strength and conditioning for netball: a needs analysis and training recommendations Strength Cond J 2017 39 4 10 21 10.1519/SSC.0000000000000287

[18] Murphy DF Connolly DA Beynnon BD Risk factors for lower extremity injury: a review of the literature Br J Sports Med 2003 37 1 13 29 10.1136/bjsm.37.1.13 12547739 PMC1724594

